# Regulation of whole-body angular momentum during human walking

**DOI:** 10.1038/s41598-023-34910-5

**Published:** 2023-05-17

**Authors:** Takuo Negishi, Naomichi Ogihara

**Affiliations:** Department of Biological Sciences, Graduate School of Science, The University of Tokyo, 7-3-1, Hongo, Bunkyo-ku, Tokyo, 113-0033 Japan

**Keywords:** Biomedical engineering, Biomechanics

## Abstract

In human walking, whole-body angular momentum (WBAM) about the body centre-of-mass is reportedly maintained in a small range throughout a gait cycle by the intersegmental cancellation of angular momentum. However, the WBAM is certainly not zero, which indicates that external moments applied from the ground due to ground reaction forces (GRFs) and vertical free moments (VFMs) counteract the WBAM. This study provides a complete dataset of the WBAM, each segmental angular momentum, and the external moments due to GRFs and VFMs during human walking. This is done to test whether (1) the three components of the WBAM are cancelled by coordinated intersegmental movements, and whether (2) the external moments due to GRFs and VFMs contribute only minimally to WBAM regulation throughout a gait cycle. This study demonstrates that WBAM is regulated in a small range not only by the segment-to-segment cancellation, but also largely through contributions by the GRFs. The magnitude of VFM is significantly smaller than the peak vertical moment generated by the GRFs; however, in the single-support phase during walking, the VFM is possibly critical for coping with the change in the vertical WBAM due to force perturbations and arm or trunk movements.

## Introduction

Appropriate control of whole-body angular momentum (WBAM) about a body centre-of-mass (COM) is critical for generating rotationally stable bipedal locomotion. Therefore, WBAM has been used as a primary measure for evaluating the risks of falls in human walking^1–11^.

With a principal component analysis (PCA) of segmental angular momentum, Herr and Popovic^12^ experimentally studied the regulation of the WBAM about the body COM in human walking. They found that throughout a gait cycle, the segment-to-segment cancellation of angular momentum is the main factor responsible for regulating the WBAM, as well as for maintaining it in a small range in all planes (up to 95%, 70%, and 80% in the sagittal, frontal, and horizontal planes, respectively). However, the WBAM is not zero; it varies in a gait cycle^12^. This indicates that the external moments applied from the ground owing to ground reaction forces (GRFs) and vertical free moments (VFMs), i.e., the torque about a vertical axis due to friction between the foot and ground, also counteract the WBAM^13–19^. This is because based on the law of mechanics, only the external moments applied to the body can change the WBAM^20^. Using the coefficient of cancellation, Bennett et al.^4^ reported that although segment-to-segment cancellation of segmental angular momenta occurred in the sagittal plane (approximately 75%), those in the frontal and horizontal planes were small (approximately 25% and 50%, respectively). This suggests that the contribution of segment-to-segment cancellation of angular momenta was relatively minor. By contrast, the three components of GRFs and VFMs highly contributed to regulating the WBAM during walking. However, only a few studies have previously investigated the relationship between the WBAM and the external moments about the body COM due to the GRFs in all three planes^5,12^. Therefore, the extent to which each of the two strategies, namely, (1) segment-to-segment cancellation of segmental angular momenta and (2) generation of external moments applied to the body from the ground, contribute to regulating WBAM is unclear.

The studies of Herr and Popovic^12^, and Silverman et al.^5^ did not provide segmental or directional breakdowns of the WBAM and the external moments due to the GRFs and VFMs. Resultingly, the extent to which each of the two strategies contributed to regulating the WBAM during human walking is difficult to determine. Therefore, this study aimed to provide a complete dataset of the WBAM, each segmental momentum, and the external moments due to GRFs and VFMs during human walking. It aimed to test whether (1) the segmental angular momenta are sufficiently cancelled out in all three planes by coordinated intersegmental movements, and whether (2) the external moments due to the GRFs and VFMs only minimally contribute to the regulation of the WBAM during a gait cycle. Improved understanding of the control of WBAM in human walking should contribute to the development of fall prevention measures in rehabilitation medicine^21^ and the understanding of human bipedal locomotion evolution and its associated derived features in the human musculoskeletal system^22^.

## Materials and methods

Ten adult males without any history of orthopaedic or neuromuscular impairments (mean [± standard deviation] age, 26.7 ± 3.5 y; height, 1.70 ± 0.04 m; weight, 61.5 ± 5.0 kg; COM height in quiet standing, 0.98 ± 0.02 m) participated in this study. Informed consent was obtained from all of them. This study was reviewed and approved by the Office for Life Science Research Ethics and Safety at the University of Tokyo. All methods were performed following the relevant guidelines and regulations.

**Figure 1:**
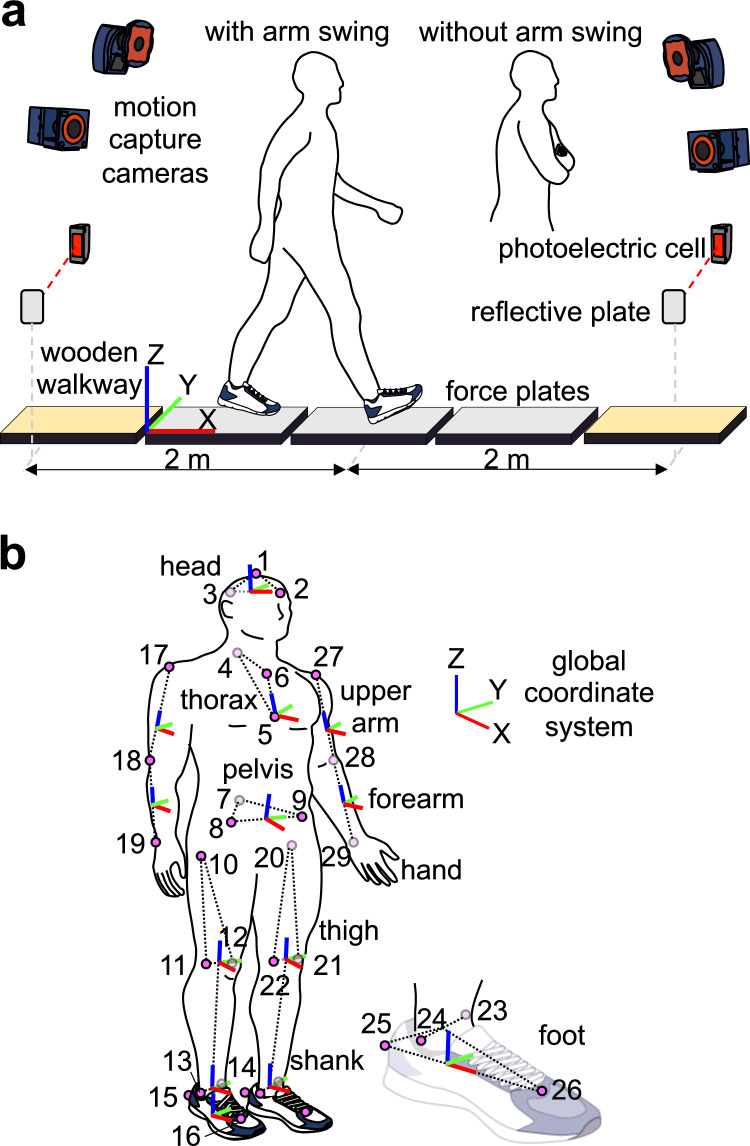
(**a**) Experimental setup and (**b**) placement of reflective markers and segment-fixed coordinate systems. A total of 29 markers are attached to: (1) the top head, (2) front head, (3) rear head, (4) 1st thoracic vertebra, (5) xiphoid process, (6) manubrium of the sternum, (7) sacrum, (8, 9) anterior superior iliac spine, (10,20) greater trochanter, (11,21) lateral knee, (12,22) medial knee, (13, 23) lateral ankle, (14, 24) medial ankle, (15, 25) heel, (16, 26) toe, (17, 27) acromion, (18, 28) elbow, and (19, 29) wrist. Markers on the toes and heels are placed on the corresponding surface positions of the shoes. The hand segment is defined as the point mass in this study; hence, a segment-fixed coordinate system is not defined for the hand segment.

The participants walked across three force plates (EFP-S-1.5KNSA13; Kyowa Dengyo, Tokyo, Japan) set in a wooden walkway (8.2-m long) with ordinary running shoes (JOG100-2; Asics, Kobe, Japan) of appropriate size (Fig. 1a). Body kinematics were recorded using a motion capture system consisting of ten cameras (MAC3D; Motion Analysis Corporation, Santa Rosa, CA, USA). A total of 29 reflective markers were attached to the body based on the modified Helen Hayes marker set (Fig. 1b)^23^, and the positions of the markers were captured at 100 Hz. The GRF signals were simultaneously recorded at 200 Hz using a universal recorder (EDX-100A; Kyowa Dengyo, Tokyo, Japan). Because arm swing during walking affects the VFM profiles^14,18,19^, the participants were first asked to walk (1) with arm swing (normal arm swing), and then (2) with their arms folded (no arm swing). The participants were instructed to step on the three force plates with their left, right, and left feet consecutively. Two pairs of photoelectric cells were placed 4 m apart across the force plates to instantaneously measure the walking speed of each trial. Trials with a walking speed within 5% of the target speed (1.1 m/s) were selected as successful trials, and five successful trials were recorded for each arm swing condition.

The gait cycle duration, stride length (horizontal distance travelled in a gait cycle), and speed were calculated using the motion-captured and force plate data. The gait cycle was defined as the interval between two successive right heel contacts. The marker data were low-pass filtered at 7 Hz using a zero-phase-shift low-pass filter^24^. No low-pass filtering was applied to the GRF signals. To quantify the three-dimensional body kinematics, a segment-fixed coordinate system was defined for each of the 13 body segments (head, thorax, pelvis, upper arms, forearms, thighs, shanks, and feet) using the attached markers (Fig. 1b). The *x*-, *y*-, and *z*-axes approximately pointed to the anteroposterior, mediolateral, and superoinferior directions, respectively. The joint angles of the neck, thoraco-pelvic, hip, knee, ankle, and shoulder were calculated as the motions of the distal segment coordinate systems with respect to the proximal coordinate systems using the *y*-*x*-*z* Euler angles. All joint angles were set to zero in a quiet standing posture.

A 15-segment whole-body model, consisting of the above 13 segments and hands defined as point masses, was used to calculate the WBAM about the body’s COM. The WBAM about the body’s COM, **L**, was calculated as the sum of the individual segment angular momenta as follows^12^.

1 $$\mathbf{L}=\sum_{i=1}^{15}{(\mathbf{c}}_{i}\times {m}_{i}{\dot{\mathbf{c}}}_{i}+{\mathbf{I}}_{i}{{\varvec{\upomega}}}_{i}),$$

where *m*_*i*_ is the mass, **I**_*i*_ is the inertia tensor about the segment’s COM, **c**_*i*_ is the COM position vector with respect to the whole-body COM, ${\dot{\mathbf{c}}}_{i}$ is the COM velocity vector with respect to the whole-body COM (time derivative of **c**_*i*_), and **ω**_*i*_ is the angular velocity vector; each one belongs to the *i*-th segment. All of these are represented in the laboratory coordinate system. The segmental masses, inertia tensors, and COM positions of each participant were estimated based on the measured marker positions and the anthropometric parameters (the ratio of the segment mass to the total body mass, the segment’s COM location along its longitudinal axis as a percentage of the segment length, and the radii of gyration of the segment as percentages of the segment length) presented in de Leva^25^ and Zatsiorsky^26^.

The net external moments about the body’s COM due to GRFs and VFMs, **M**, can be calculated as follows.

$$\mathbf{M}={\mathbf{r}}_{L}\times {\mathbf{F}}_{L}+{\mathbf{r}}_{R}\times {\mathbf{F}}_{R}+{{\varvec{\uptau}}}_{L}+{{\varvec{\uptau}}}_{R}$$

2 $$=\left[\begin{array}{l}{r}_{Ly}{F}_{Lz}-{r}_{Lz}{F}_{Ly}+{r}_{Ry}{F}_{Rz}-{r}_{Rz}{F}_{Ry}\\ {r}_{Lz}{F}_{Lx}-{r}_{Lx}{F}_{Lz}+{r}_{Rz}{F}_{Rx}-{r}_{Rx}{F}_{Rz}\\ {r}_{Lx}{F}_{Ly}-{r}_{Ly}{F}_{Lx}+{r}_{Rx}{F}_{Ry}-{r}_{Ry}{F}_{Rx}+{\tau }_{L}+{\tau }_{R}\end{array}\right],$$

where ${\mathbf{r}}_{L}={({r}_{Lx}, {r}_{Ly},{r}_{Lz})}^{T}$ and ${\mathbf{r}}_{R}={({r}_{Rx},{r}_{Ry},{r}_{Rz})}^{T}$ are the vectors pointing to the left and right centres-of-pressure (COPs) from the body’s COM, respectively; ${\mathbf{F}}_{L}={({F}_{Lx},{F}_{Ly},{F}_{Lz})}^{T}$ and ${\mathbf{F}}_{R}={({F}_{Rx},{F}_{Ry},{F}_{Rz})}^{T}$ are the GRF vectors applied to the left and right feet, respectively; and ${{\varvec{\uptau}}}_{L}={(0, 0, {\tau }_{L})}^{T}$ and ${{\varvec{\uptau}}}_{R}={(0, 0, {\tau }_{R})}^{T}$ are the VFM vectors acting on the left and right feet, respectively. In theory, $\dot{\mathbf{L}}=\mathbf{M}.$ However, this equation is not usually satisfied in experimental analyses primarily owing to the errors associated with estimating the masses, inertia tensors, and COM positions of the body segments. To account for this error, we calculated the residual **ε**, as follows.

3 $${\varvec{\upvarepsilon}}=\dot{\mathbf{L}}-\mathbf{M},$$

For comparisons, the GRFs were normalized by the body mass × the gravitational acceleration. The WBAM was normalized by the product of the body mass, COM height, and walking velocity^12^. The external moments about the body’s COM, **M**, and the rate of change of WBAM were normalized by the product of the body mass, gravitational acceleration, and COM height^12^.

To quantify the ratio of the total amount of segmental angular momenta that cancelled each other out of the total magnitudes of segmental angular momenta, the so-called coefficient of cancellation around the *j-*th axis (*j* = *x*, *y*, *z*), *κ*_j_, was calculated, as follows^4,27^.

4 $${\kappa }_{j}=\frac{{A}_{j}-{B}_{j}}{{A}_{j}}, {A}_{j}={\sum }_{i=1}^{N}\left|{L}_{ij}\right|, {B}_{j}=\left|{\sum }_{i=1}^{N}{L}_{ij}\right|,$$

where *L*_*ij*_ is the angular momentum of the *i-*th segment around the *j-*th axis, *A*_*j*_ is the total magnitude of segmental angular momentum, and *B*_*j*_ is the total amount of angular momentum generated by the external moments. Therefore, ${A}_{j}-{B}_{j}$ represents the total amount of angular momenta that cancelled each other out due to segment-to-segment cancellation. The coefficient values of one and zero thus indicate that 100% and 0% segment-to-segment cancellation of angular momenta was achieved, respectively. However, in this study, not only the ratios (coefficients of cancellation) but also the absolute magnitudes of ${A}_{j}, {B}_{j},$ and ${A}_{j}-{B}_{j}$ were analysed to account for the difference in the magnitudes of the segmental angular momenta around the three directions. In addition, to confirm whether the WBAM about the body COM during walking could be described by a small number of principal components (PCs), PCA was performed on the angular momentum profiles of 15 segments, as described in Herr and Popovic^12^.

To test for significant differences in the maximum and minimum peak values of joint angles, GRF, VFM, WBAM, segmental angular momenta, coefficients of cancellation, and external moments between the two arm swing conditions, two-tailed paired t-tests were performed. If the normality or homogeneity was violated using the Shapiro–Wilk normality test, the Wilcoxon signed-rank test was used. The statistical significance level was set at *p* < 0.05. All statistical analyses were performed using R version 4.1.2^28^.

## Results

Mean gait cycle duration, stride length, and speed were essentially identical between gait with and without arm swing: 1.20 ± 0.05 s and 1.20 ± 0.07 s, respectively, for a cycle duration; 1.34 ± 0.06 m and 1.35 ± 0.06 m, respectively, for stride length; and 1.10 ± 0.02 m/s and 1.11 ± 0.02 m/s, respectively, for speed. This indicated that no differences in the spatiotemporal parameters were found between the two conditions. The Froude number equivalent to the present target speed was 0.14.

**Figure 2:**
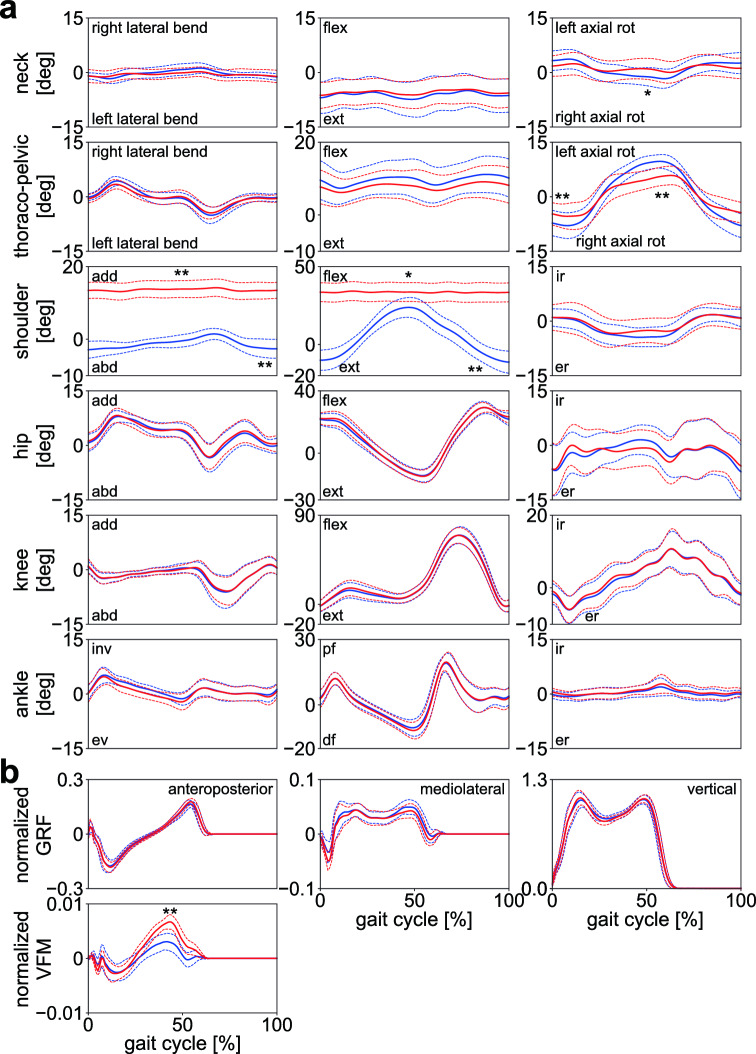
(**a**) Mean joint angle and (**b**) normalized GRF and VFM profiles during walking with (blue) and without (red) arm swing. Corresponding dashed lines represent standard deviations. Asterisks indicate statistical differences of the maximum or minimum values (*: *p* < 0.05. **: *p* < 0.01).

The mean joint angle profiles were identical between the two conditions (Fig. 2a); however, the ranges of axial rotations of the neck and thoraco-pelvic joints were significantly larger in gait with arm swing than in gait without it. The shoulder joint angle was constant throughout a gait cycle because the arms were folded, and hence, arm swing was restricted. The mean GRF profiles were also identical between the two conditions; however, the VFMs were significantly different, with the magnitude being significantly reduced in the second half of the stance phase due to arm swing (Fig. 2b; Supplementary Table S1).

**Figure 3:**
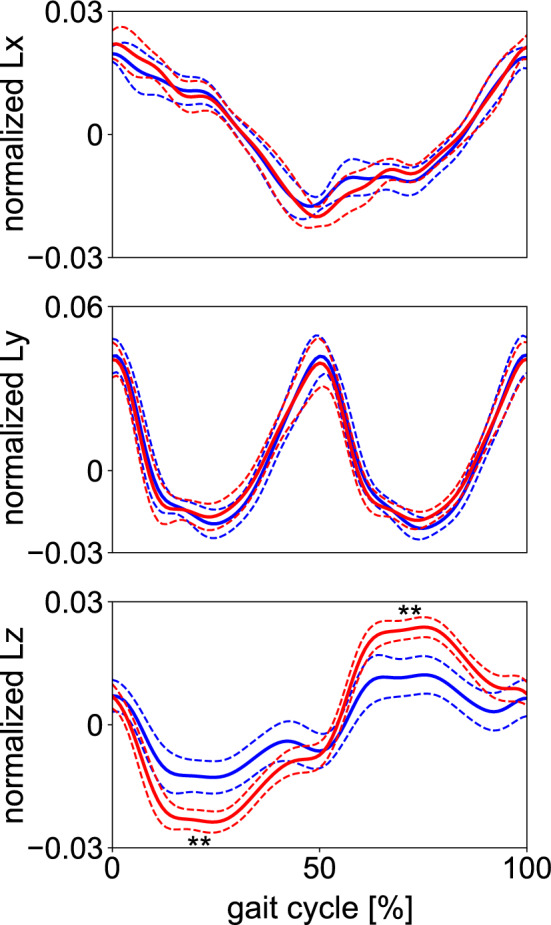
Mean normalized WBAM profiles about the body COM during walking with (blue) and without (red) arm swing in the frontal, sagittal, and transverse planes (Lx, Ly and Lz, respectively). Corresponding dashed lines represent standard deviations. Asterisks indicate statistical differences of the maximum or minimum values (*: *p* < 0.05. **: *p* < 0.01).

**Figure 4:**
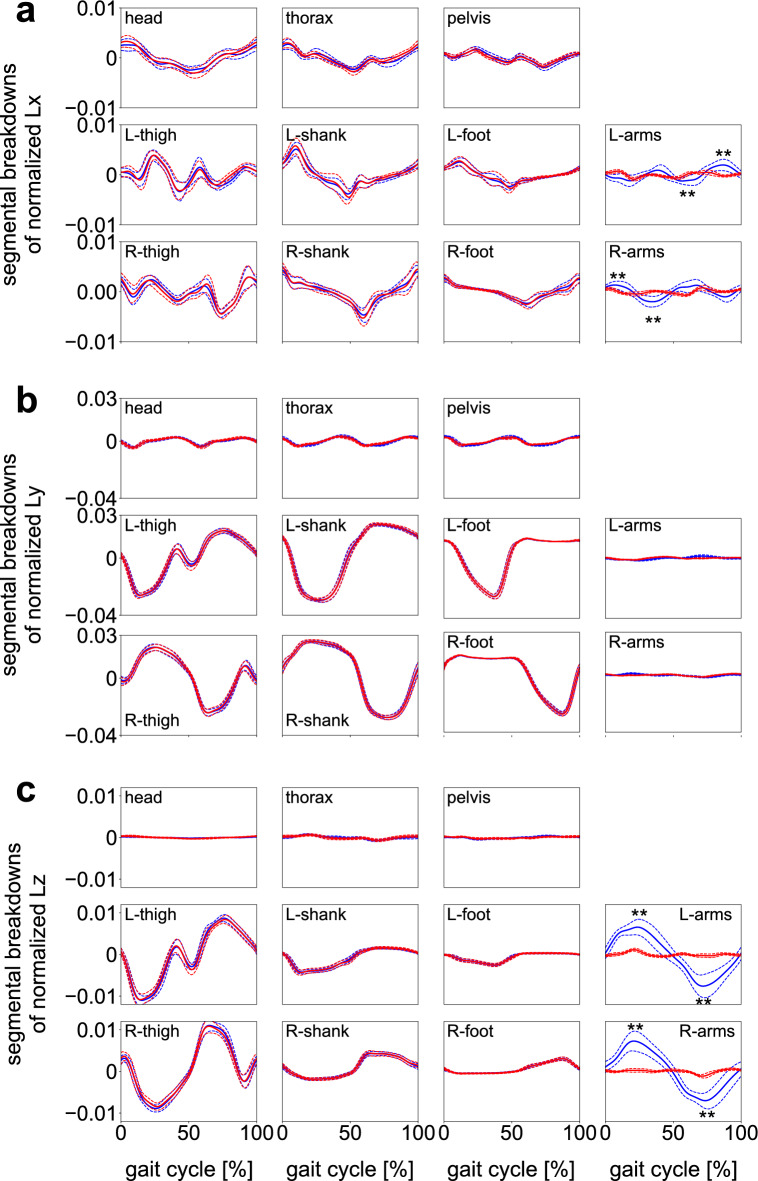
Segmental breakdowns of the mean normalized WBAM profiles in the frontal plane (Lx, about the anteroposterior axis, (**a**), sagittal plane (Ly, about the mediolateral axis, (**b**), and horizontal plane (Lz, about the vertical axis, (**c**) during walking with (blue) and without (red) arm swing. The angular momenta of the upper arm, forearm, and hand are consolidated for simplicity. Corresponding dashed lines represent standard deviations. Asterisks indicate statistical differences of the maximum or minimum values (*: *p* < 0.05. **: *p* < 0.01).

Figure 3 compares the mean WBAM profiles between the two conditions. The WBAM profiles were nearly identical in the frontal and sagittal planes (Lx, Ly); however, the amplitude of the WBAM in the transverse plane (Lz) was significantly larger in gait without arm swing (Fig. 3; Supplementary Table S1). Figure 4 depicts the segmental breakdowns of the WBAM in the frontal, sagittal, and transverse planes. Frontally, the angular momenta of the head, thorax, shanks, and feet fluctuated nearly in phase, constituting the main component of the WBAM (Fig. 4a). However, no segmental angular momentum fluctuated out-of-phase, thus indicating that no clear segment-to-segment cancellations of angular momentum occurred in the frontal plane during walking. A slight difference was observed in the angular momenta of the arms between gait with and without arm swing; however, this had only minor effects on the frontal WBAM. Sagittally, the WBAM was primarily composed of the angular momenta of the thighs, shanks, and feet (Fig. 4b). The angular momenta of the right thigh, shank, and foot fluctuated in phase; however, the three segments of the right and left legs moved out-of-phase, thereby enabling effective intersegmental cancellation of angular momentum. The angular momenta of the trunk segments and arms had only minor effects on the sagittal WBAM. Transversely, the angular momenta of the thighs that fluctuated in phase were the main component of the WBAM (Fig. 4c). This was counteracted by the angular momenta of the arms, which fluctuated out-of-phase with the thigh segments, resulting in segment-to-segment cancellation of angular momentum. Thus, a significantly smaller magnitude of the WBAM was obtained in gait with arm swing (Fig. 3; Supplementary Table S1). Additionally, we observed that the angular momenta of the thorax and pelvis segments fluctuated out-of-phase; however, their magnitudes were much smaller than the angular momenta of other segments (Fig. 4c).

**Figure 5:**
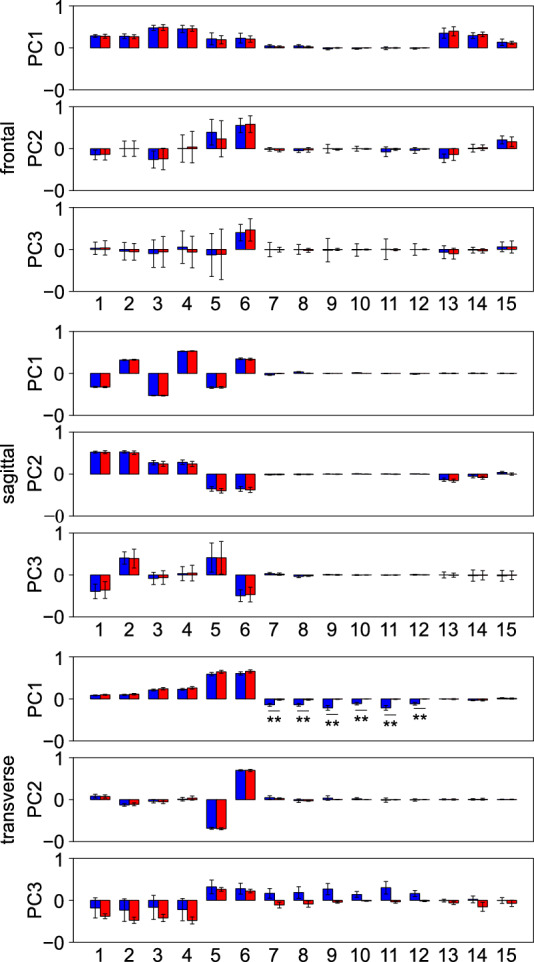
Mean coefficients of the first three angular momentum principal components in the frontal, sagittal, and horizontal planes during walking with (blue) and without (red) arm swing. The number corresponds to the following segments: (1, 2) left and right foot, (3, 4) left and right shank, (5, 6) left and right thigh, (7, 8) left and right upper arm, (9, 11) left and right forearm, (10, 12) left and right hand, (13) head, (14) thorax, and (15) pelvis.

The PCA of the angular momentum profiles of 15 segments revealed that for gait with arm swing, the first three PCs accounted for 91.4 ± 2.1%, 96.7 ± 0.9%, and 99.0 ± 0.4% of the total variance of angular momentum in the frontal, sagittal, and transverse planes, respectively. For gait without arm swing, the first three PCs accounted for 91.9 ± 2.1%, 97.2 ± 0.7%, and 99.4 ± 0.2% of the total variance of angular momentum in the frontal, sagittal, and transverse planes respectively. These results indicate that the WBAM about the body COM during walking could be described by a small number of PCs. The first three PCs in the three planes, presented in Fig. 5, were almost identical between gait with and without arm swing, except for the coefficients corresponding to the arm segments. The coefficients of the first PC were distributed in both positive and negative directions in the sagittal and transverse planes, thus indicating that a certain degree of segment-to-segment cancellation of angular momentum took place in these two planes. In the frontal plane, however, the coefficients of the first PC were all positive, thus suggesting no meaningful segment-to-segment cancellation of angular momentum.

**Figure 6:**
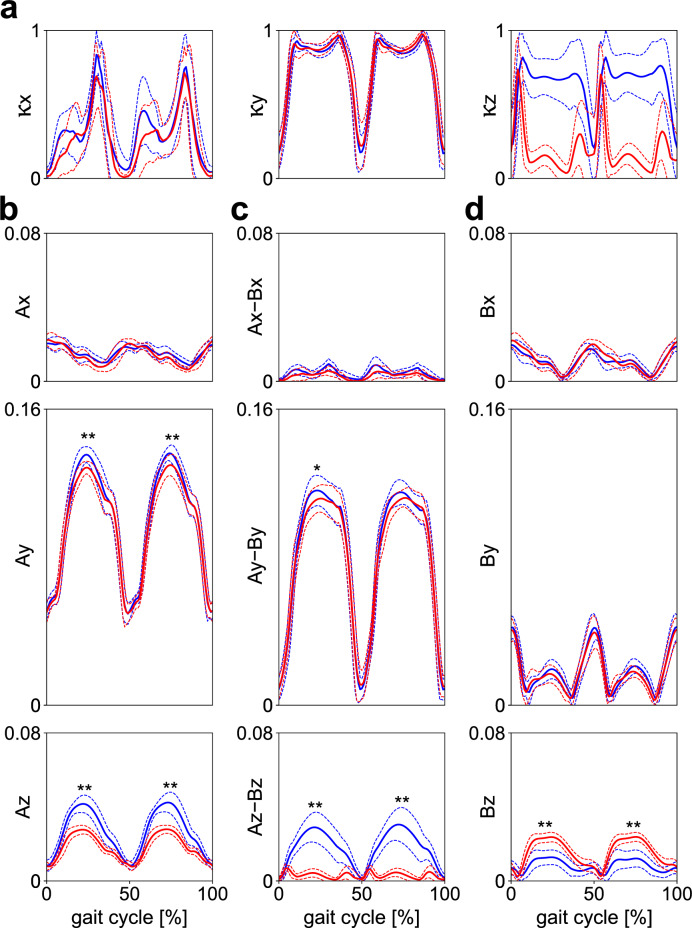
(**a**) Mean profiles of the coefficient of cancellation of segmental angular momenta, (**b**) the total magnitude of normalized segmental angular momentum, (**c**) its component that is cancelled owing to segment-to-segment cancellation, and (**d**) that generated by the external moments in the frontal, sagittal, and horizontal planes during walking with (blue) and without (red) arm swing. The coefficient values of one and zero indicate that perfect and no segment-to-segment cancellation of angular momenta were achieved, respectively. Corresponding dashed lines represent standard deviations. Asterisks indicate statistical differences of the maximum or minimum values (*: *p* < 0.05. **: *p* < 0.01).

The coefficients of cancellation profiles in the three planes are presented in Fig. 6a. The mean coefficients of cancellation were calculated to be 0.320 ± 0.056, 0.743 ± 0.032, and 0.636 ± 0.122 in the frontal, sagittal, and transverse planes, respectively, for gait with arm swing. For gait without swing, they were 0.264 ± 0.065, 0.756 ± 0.031, and 0.196 ± 0.101, respectively. These values indicated that the WBAM was regulated by segment-to-segment cancellation in the sagittal plane, but not in the frontal plane. In the transverse plane, the coefficient was significantly larger in gait with arm swing (0.636 ± 0.122 vs 0.196 ± 0.101, *p* < 0.01) (Fig. 6a; Supplementary Table S1), indicating that arm swing largely contributed to the intersegmental cancellation of angular momentum. Comparisons of the total magnitude of segmental angular momentum, its component that cancelled each other owing to segment-to-segment cancellation, and the rest of the net moment generated by the GRFs and VFMs (Fig. 6b–d) also demonstrate that the WBAM was regulated by segment-to-segment cancellation in the sagittal plane, but not in the frontal plane. The largest magnitude of angular momentum by the external moments was generated in the sagittal plane, even though the contribution of segment-to-segment cancellation of angular momentum was the largest in it.

**Figure 7:**
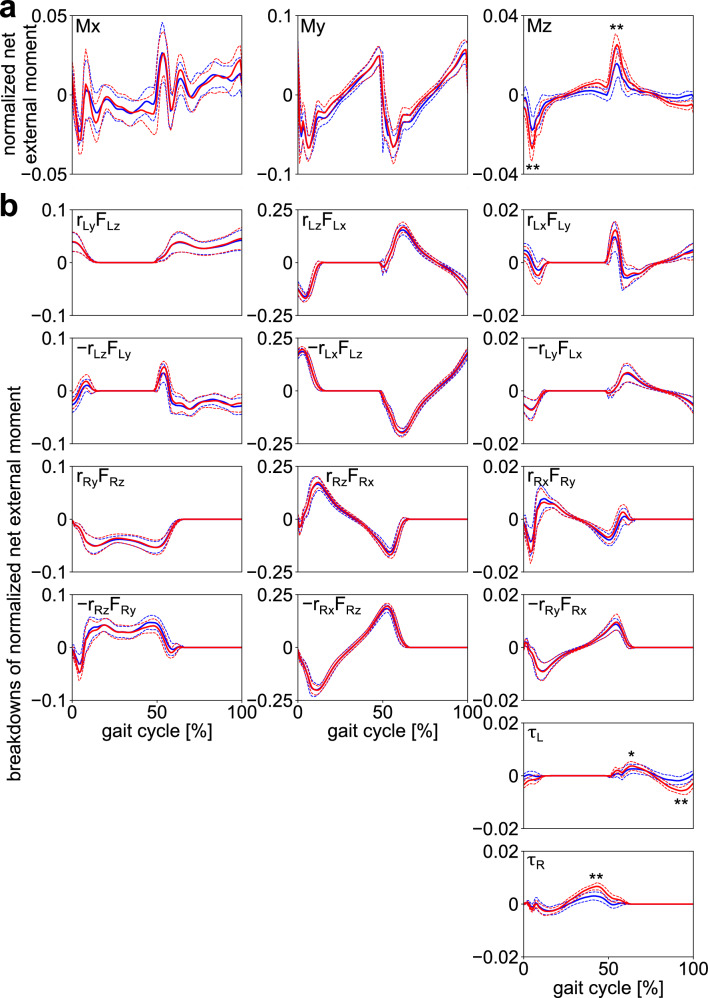
(**a**) Mean normalized external moments about the body COM applied from the ground and (**b**) their breakdowns during walking with (blue) and without (red) arm swing. Corresponding dashed lines represent standard deviations. Asterisks indicate statistical differences of the maximum or minimum values (*: *p* < 0.05. **: *p* < 0.01).

Figure 7a,b display the net external moments applied to the body COM due to GRFs and VFMs, and the breakdowns of the net external moments, respectively, in the frontal, sagittal, and transverse planes. The fluctuation of the net external moment was the largest in the sagittal plane. It sharply decreased soon after heel contact owing to the moment in a backward-leaning direction by the vertical GRF of the leading leg (F_Rz_) and the propulsive GRF of the trailing leg (F_Lx_). Subsequently, it gradually increased to generate the moment in the forward-leaning direction until the heel contact of the contralateral leg (Fig. 7a). The waveforms and amplitudes of the external moments generated by two components of the GRF (F_x_ and F_z_) were almost equal, but opposite, indicating that the external moments generated by the two components of the GRF cancelled each other (Fig. 7b). Therefore, the amplitudes of the breakdowns (Fig. 7b) were much larger than that of the corresponding net external moment (Fig. 7a). The net external moments in the frontal and transverse planes were comparatively small in amplitude. However, relatively large moments were generated during the double-support phase (Fig. 7a), owing to the laterally-directed GRF (F_y_) applied to the feet in the early stance (double-support) phase. Again, the waveforms and amplitudes of the external moments generated by two components of the GRFs (F_y_ and F_z_ and F_x_ and F_y_ in the frontal and sagittal planes, respectively) were roughly identical but only the sign was opposite, thus indicating that they cancelled out each other in all three planes (Fig. 7b).

The amplitude of the VFM (${\uptau }_{L}, {\uptau }_{R}$) was much smaller than the vertical external moments due to the GRF (${\mathbf{r}}_{L}\times {\mathbf{F}}_{L}, {\mathbf{r}}_{R}\times {\mathbf{F}}_{R}$) (Fig. 7b). The peak magnitudes of the external moment in the transverse plane were significantly larger in gait without arm swing (Fig. 7a; Supplementary Table S1) because significantly larger VFMs were applied to the feet in this condition (Fig. 7b; Supplementary Table S1).

**Figure 8:**
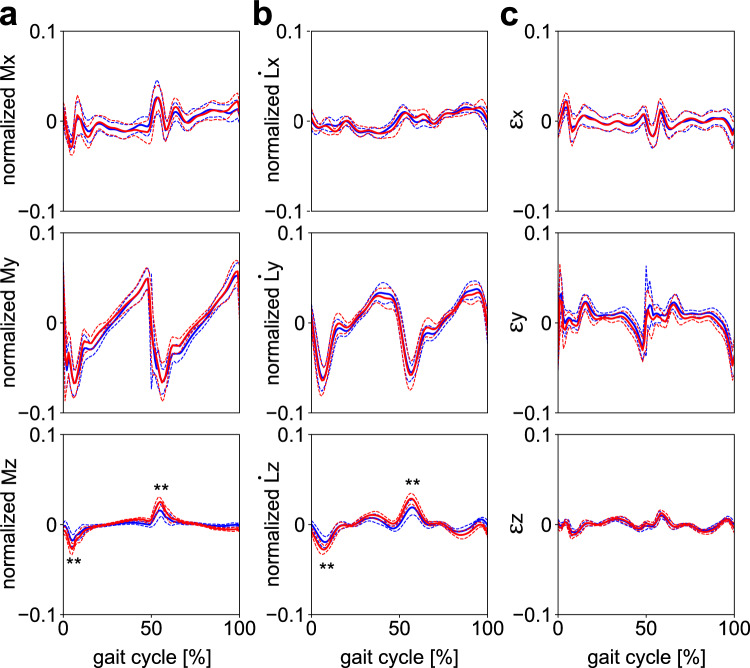
(**a**) Mean normalized external moments about the body COM applied from the ground $\mathbf{M}$, (**b**) the rate of the change of the WBAM $\dot{\mathbf{L}}$, and (**c**) the residuals between the external moments about the body COM applied from the ground and the rate of the change of the WBAM ${\varvec{\upvarepsilon}}$ during walking with (blue) and without (red) arm swing, in the frontal, sagittal, and transverse planes. Corresponding dashed lines represent standard deviations. Asterisks indicate statistical differences of the maximum or minimum values (*: *p* < 0.05. **: *p* < 0.01).

Figure 8 compares $\dot{\mathbf{L}},\mathbf{M},$ and ${\varvec{\upvarepsilon}}$ to assess the reliability of the quantification of the WBAM and external moments, considering possible errors associated with the estimation of the masses, inertia tensors, and COM positions of the body segments. The waveforms of $\dot{\mathbf{L}}$ and $\mathbf{M}$ resembled each other, and in the stance phase, ${\varvec{\upvarepsilon}}$ was found to be maintained near zero in all planes. However, relatively large residuals were observed in the double support phase, particularly in the sagittal plane.

## Discussion

This study demonstrated that segment-to-segment cancellation does not necessarily regulate the WBAM about the body COM to a small range during human walking. This is contrary to the conventional understanding that the WBAM is well-regulated and maintained in a small range in all planes due to segment-to-segment cancellation of angular momentum^12^. In the sagittal plane, the WBAM was found to be primarily minimized by segment-to-segment cancellation; this was also true in the transverse plane when walking with normal arm swing, as observed in previous studies^4,27^. However, in the frontal plane, the contribution of segment-to-segment cancellation remained minimal. This is also true in the transverse plane when the arm swing is restrained. If a perfect cancellation of angular momentum had been achieved in a gait cycle due to intersegmental cancellation, the WBAM would have been zero. However, this and previous studies^4,12,29^ demonstrated that the WBAM was not zero and varied with time, thus indicating that segmental angular momenta were not perfectly cancelled due to segment-to-segment cancellation.

Although the segment-to-segment cancellation of angular momentum had the highest coefficient of cancellation (indicating the ratio of cancellation) in the sagittal plane, the absolute magnitude of the external moment that should have been explicitly generated was also the largest in this plane (Fig. 6d). This is because the magnitude of the WBAM was relatively larger in the sagittal plane than in the other two planes (Fig. 3). Therefore, for generating stable bipedal locomotion, the WBAM should be regulated in all three planes by the external moments applied from the ground due to GRFs and VFMs. However, we also confirmed that the WBAM was partly regulated by segment-to-segment cancellation, as suggested previously^4,12^.

In the frontal, sagittal, and transverse planes, the waveforms and amplitudes of the external moment generated by the two components of the GRF vector (i.e., Fy and Fz, Fx and Fz, and Fx and Fy) were roughly identical. However, the signs were opposite (Fig. 7b). Hence, they naturally cancelled each other out, which was why relatively small external moments were applied around the whole-body COM. The GRF vectors did not pass exactly on the whole-body COM in the sagittal plane. Instead, they systematically passed above it to generate the moment to balance the WBAM, as reported previously^30–32^. Hence, the WBAM largely deviated from zero (Fig. 3). In the frontal plane, the GRF vectors should have passed considerably close to the COM because the net external moments applied to the whole-body COM were almost zero. In the transverse plane, the GRF vectors should also have passed considerably close to the COM, particularly in the single-support phase, because the net external moments applied to the whole-body COM were almost zero during the single-support phase (Fig. 7a). However, in the double support phase, comparatively large moments were applied owing to the laterally-directed GRF vectors applied in the early stance phase. Essentially, the WBAM around the vertical axis was not regulated in the single-support phase, and it was regulated intermittently in the double-support phase by the relatively small and brief generation of peak lateral GRFs during the early stance phase (Fig. 2b). This explains why the WBAM around the vertical axis fluctuated roughly as a rectangular wave (Fig. 3). Therefore, this study indicated that the small lateral peak of the mediolateral GRF in the early stance phase might play a crucial role in the regulation of the vertical WBAM during walking.

Moreover, the angular momenta of the thorax and pelvis segments fluctuated out-of-phase (Fig. 4c), reportedly contributing to reducing the vertical WBAM^12,33–35^. However, this study found that their magnitudes were much smaller than those of other segments (Fig. 4c). The intersegmental cancellation of angular momentum between the thorax and pelvis has been suggested as significant for the generation of efficient bipedal walking. However, this study found that the main components of the vertical WBAM were the angular momenta of the arms and legs but not the thorax and pelvis, as also suggested by previous studies^4,12,35^.

In addition, in this study, the magnitude of the VFM was much smaller than the external moments generated by the GRFs in the transverse plane. This indicated that the relative contribution of the VFM on the regulation of the WBAM during human walking was minor, particularly during normal gait with arm swing (Fig. 7b). However, this moment is essential to prevent the foot from rotating around the vertical axis. The VFM is the frictional moment generated between the plantar surface of the foot and the ground; therefore, the VFM can compensate for changes in vertical WBAM that might have occurred due to external perturbations or changes in arm and trunk movements. This is true unless the vertical WBAM does not exceed the limiting condition of static friction. This compensation is demonstrated by the larger VFM in gait without arm swing (Fig. 2b). Therefore, the larger the limit of static friction, the larger the ability to regulate the vertical WBAM during gait. The human foot possesses a plantigrade foot with an enlarged calcaneus, the tuberosity of which points posteroinferiorly, thereby allowing prominent heel-strike^36–38^. In addition, the human foot lost prehensile capability, and hence, the phalanges were relatively shorter than those of other non-human primates^39,40^. Furthermore, the nonopposable hallux was aligned in parallel with the other four digits^41,42^. These morphological characteristics enlarge the area of the plantar surface and potentially increase the ability to generate a large VFM. If the plantar surface of the foot is larger, the moment arm of the horizontal GRF with respect to the COP is larger, and the limiting condition of static friction should be larger^43^. The surface to walk on could be very small as people can walk on stilts, but generating a large VFM is difficult on small surfaces because the moment arm is very small^44^. Therefore, we hypothesize that the plantigrade foot with a large plantar surface was beneficial for stabilization of bipedal locomotion and might have evolved as an adaptation to rotationally stabilize bipedal locomotion around the vertical axis over the course of human evolution. However, this possibility must be verified in future studies.

This study had several limitations. First, the accuracy of estimation of the segment mass, COM position, and moments of inertia necessary for calculating the WBAM could improve with medical imaging. Herein, these inertial parameters were calculated based on the average values of the corresponding cadaver samples. However, they vary among people^26^, thus resulting in large residuals ${\varvec{\upvarepsilon}}$ in this study (Fig. 8c). For a more rigorous estimation of the inertial parameters, subject-specific inertial parameters could be obtained using medical imaging, such as magnetic resonance imaging and computed tomography^45^. Although we believe that this inaccuracy does not affect the main conclusions of this study, the current findings should be confirmed based on subject-specific inertial parameters. Second, the participants of this study were all adult males, although statistically, significant sex differences have been reported to exist in whole-body kinematics during walking^46^. Therefore, the current findings should also be confirmed with female participants. However, the absolute differences in the joint angles between the two were negligible. Therefore, we believe this is not a major limitation of this study. Third, the target walking speed was relatively low in the present study. Fourth, the order of the two conditions was not randomized. Although we do not believe these affect the results of this study, future studies should dispel such concerns.

## Supplementary Information

Supplementary Information.

## Data Availability

The datasets generated during and/or analysed during the current study are available from the corresponding author on reasonable request.

## Code availability

The computer code used for this study is available from the corresponding author upon reasonable request.
